# Immune cell-derived exosomes in cancer: double-edged mechanisms of antitumor immunity and tumor progression

**DOI:** 10.3389/fimmu.2026.1937923

**Published:** 2026-09-07

**Authors:** Maryam Firouzi, Angelina Akomea, Mohammad Hasan Jamei, Greg M. Harris, Dongin Kim

**Affiliations:** 1 Department of Pharmaceutical Sciences, University of Oklahoma Health Campus, Oklahoma City, OK, United States; 2 Department of Biomedical Engineering, College of Engineering and Applied Science, University of Cincinnati, Cincinnati, OH, United States; 3 Department of Chemical and Environmental Engineering, College of Engineering and Applied Science, University of Cincinnati, Cincinnati, OH, United States

**Keywords:** immune cell-derived exosomes, antitumor activation, cancer immunomodulation, cancer signaling pathways, tumor microenvironment, tumor progression

## Abstract

Immune cell-derived exosomes are important mediators of intercellular communication within the tumor microenvironment. Their biological effects are influenced by the immune-cell source and activation state, vesicular cargo, recipient-cell type, and tumor context. This review examines immune cell-derived exosomes as bidirectional regulators of cancer-associated signaling, with emphasis on their antitumor and protumor mechanisms rather than classification by parent cell type alone. Antitumor exosomes can enhance antigen presentation, activate natural killer cell and T-cell responses, reprogram tumor-associated macrophages, reduce immune-checkpoint signaling, induce tumor-cell death, and suppress tumor growth and metastasis. Conversely, exosomes released by regulatory T cells (Tregs), myeloid-derived suppressor cells (MDSCs), tumor-associated macrophages (TAMs), and other immune populations may promote immune suppression, tolerogenic reprogramming, checkpoint-mediated immune escape, invasion, metastasis, and treatment resistance. These effects are mediated through the transfer of proteins, receptors, enzymes, cytokine-related molecules, microRNAs, long non-coding RNAs, and other regulatory cargo that modulate signaling networks such as NF-κB, MAPK/ERK, PI3K-AKT-mTOR, STAT3, PD-1/PD-L1, apoptotic, and β-catenin/HIF-1α-associated pathways. Despite their therapeutic and diagnostic potential, clinical translation remains limited by extracellular-vesicle heterogeneity, incomplete identification of functional cargo, inconsistent isolation and characterization methods, manufacturing challenges, storage instability, and difficulties in tracking vesicles *in vivo*. Future studies should aim to define the relationships among parent-cell state, exosomal cargo, recipient-cell identity, and downstream biological responses. Additionally, integrating experimental data with computational and artificial-intelligence-based approaches may support single-vesicle classification, inference of EV cellular origin, and the development of more reproducible exosome-based cancer therapies.

## 1 Introduction

**Figure 1:**
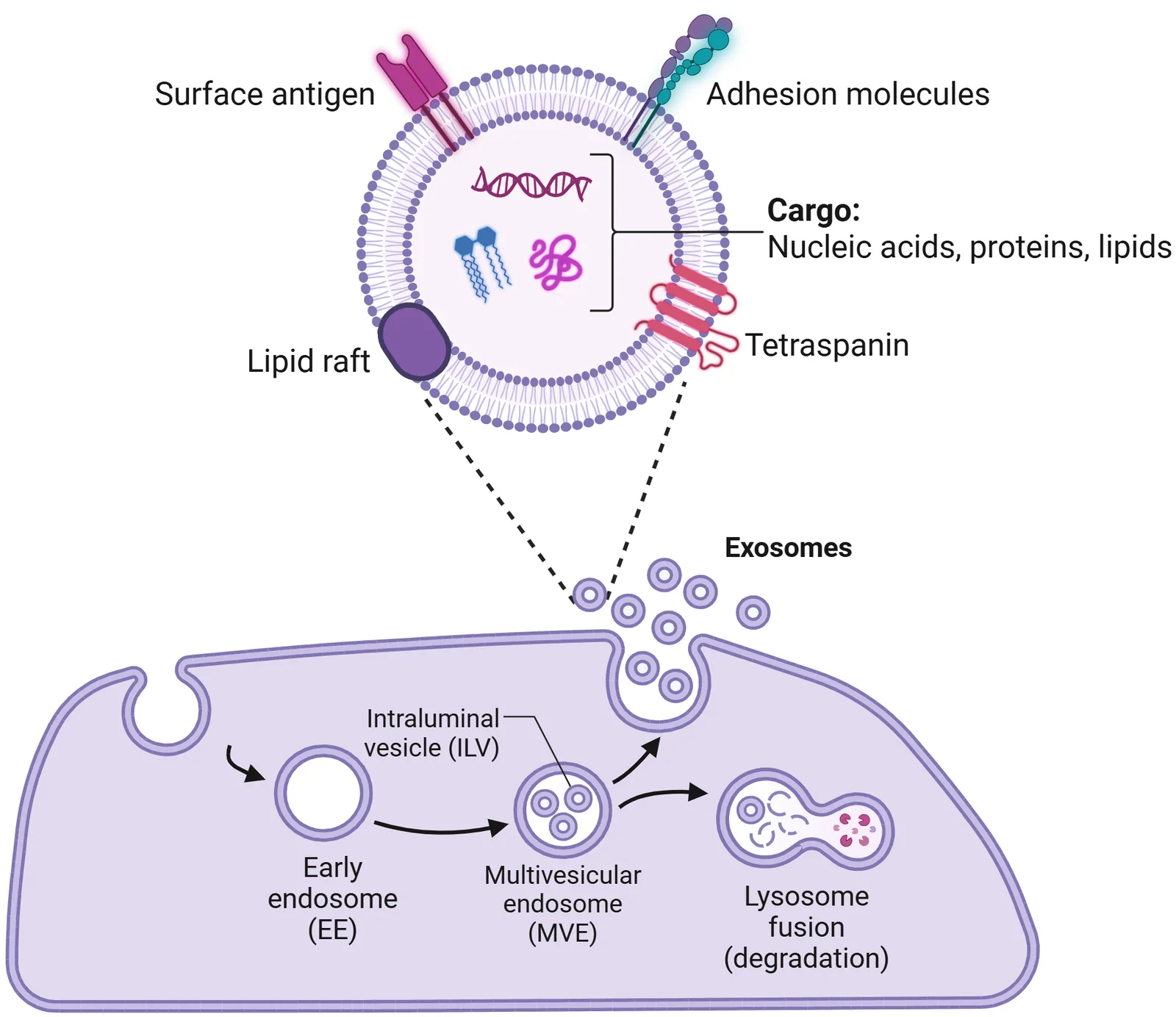
Exosome biogenesis, release, and molecular composition. Exosomes are generated through inward budding of the endosomal membrane, forming intraluminal vesicles within multivesicular endosomes. These vesicles are either degraded following lysosomal fusion or released as exosomes when multivesicular endosomes fuse with the plasma membrane. Exosomes carry nucleic acids, proteins, and lipids and display surface molecules such as tetraspanins, adhesion molecules, antigens, and lipid-raft-associated components.

Exosomes, a subclass of small extracellular vesicles (sEVs) are secreted by almost all cell types including immune cells and tumor cells (1). They are typically nanosized vesicles generated through the endosomal pathway. During exosome biogenesis, early endosomes mature into multivesicular bodies (MVBs), which contain intraluminal vesicles that are released as exosomes when MVBs fuse with the plasma membrane (2). After they are released into the extracellular environment, exosomes participate in cell-to-cell communication. These vesicles are usually found in mammalian peripheral blood as well as human urine, saliva, semen, breast milk, cerebrospinal fluid, amniotic fluid, and bronchoalveolar fluid (3, 4). They carry molecules such as proteins, lipids, mRNAs, miRNAs and other non-coding RNAs. Their biological effects mostly depend on the cell of origin, the molecular components they carry, the activation state, and the physiological or pathological state of the parent cell. Commonly identified exosomal markers for exosome characterization include tetraspanins CD9, CD63, and CD81, as well as proteins such as Hsp70 (**Figure 1**) (4).

In this review, the term exosome is used predominantly because it is the term used in most of the primary studies discussed. However, extracellular vesicles (EVs) are used as the broader term when referring to heterogeneous vesicle populations, methodological considerations, or studies that use EV or small EV (sEV) terminology. Consistent with recommendations from the International Society for Extracellular Vesicles (ISEV), biogenesis-based terms such as exosome should be interpreted cautiously when endosomal origin has not been directly demonstrated. Accordingly, when discussing individual studies, we generally retain the terminology used by the original authors (5). Moreover, although exosomes can carry diverse molecular components, including lipids, proteins, and nucleic acids, the mechanistic studies discussed in this review have predominantly characterized protein- and RNA-mediated effects.

In cancer, exosomes contribute to communication within the tumor microenvironment and can influence tumor progression, immune regulation and therapeutic response (6). Tumor-derived exosomes are associated with pro-tumor functions such as immune suppression, angiogenesis, metastasis and the transfer of tumor-promoting molecules. In contrast, immune cell-derived exosomes can exert either antitumor or protumor effects within the tumor microenvironment. Exosomes released by dendritic cells, macrophages, natural killer cells, T cells, B cells, and myeloid-derived suppressor cells (MDSCs) can regulate immune activation, antigen presentation, cytotoxic responses, inflammation, and immune suppression (4) Through the transfer of signaling proteins, receptors, cytokine-related molecules, and regulatory RNAs, these vesicles can modulate inflammatory signaling, immune-checkpoint regulation, cell-survival pathways, and tumor-immune interactions. Immune cell-derived exosomes therefore represent an important link between immune communication and cancer-associated signaling, with potential relevance to diagnostic, immunotherapeutic, and drug-delivery strategies (3, 7–9).

Several reviews have summarized the roles of immune cell-derived exosomes according to their parent cell type in the tumor microenvironment and inflammatory diseases (3, 8, 9). This cell-type-based framework has been useful for understanding the functions of exosomes released from T cells, NK cells, macrophages, dendritic cells, and other immune cell types. However, a mechanism-focused organization can facilitate comparison of shared or contrasting molecular processes across different immune-cell sources and tumor contexts. This is particularly relevant because exosomes from the same broad immune-cell population may produce different biological outcomes depending on donor-cell activation state, cargo composition, recipient-cell identity, and the signaling state of the recipient cell. Conversely, exosomes from different immune-cell sources may converge on similar antitumor or protumor mechanisms. Expanding on previous cell-type-based reviews, we therefore organize the literature according to antitumor and protumor mechanisms rather than parent cell type alone. Antitumor mechanisms include immune activation and cytokine remodeling, myeloid reprogramming, checkpoint reversal, cytotoxic and death-receptor signaling, and inhibition of tumor progression and metastasis. Protumor mechanisms include immune suppression and tolerogenic reprogramming, checkpoint-linked immune escape, metabolic reprogramming, and signaling that promotes tumor-cell survival, invasion, metastasis, and treatment resistance. We further examine how these exosomes regulate signaling pathways and interconnected cellular responses within the tumor microenvironment and evaluate the biological factors that influence whether they exert antitumor or protumor effects, together with the methodological challenges that affect interpretation of these findings and their translational potential.

## 2 Immune cell-derived exosomes in the tumor microenvironment

**Figure 2:**
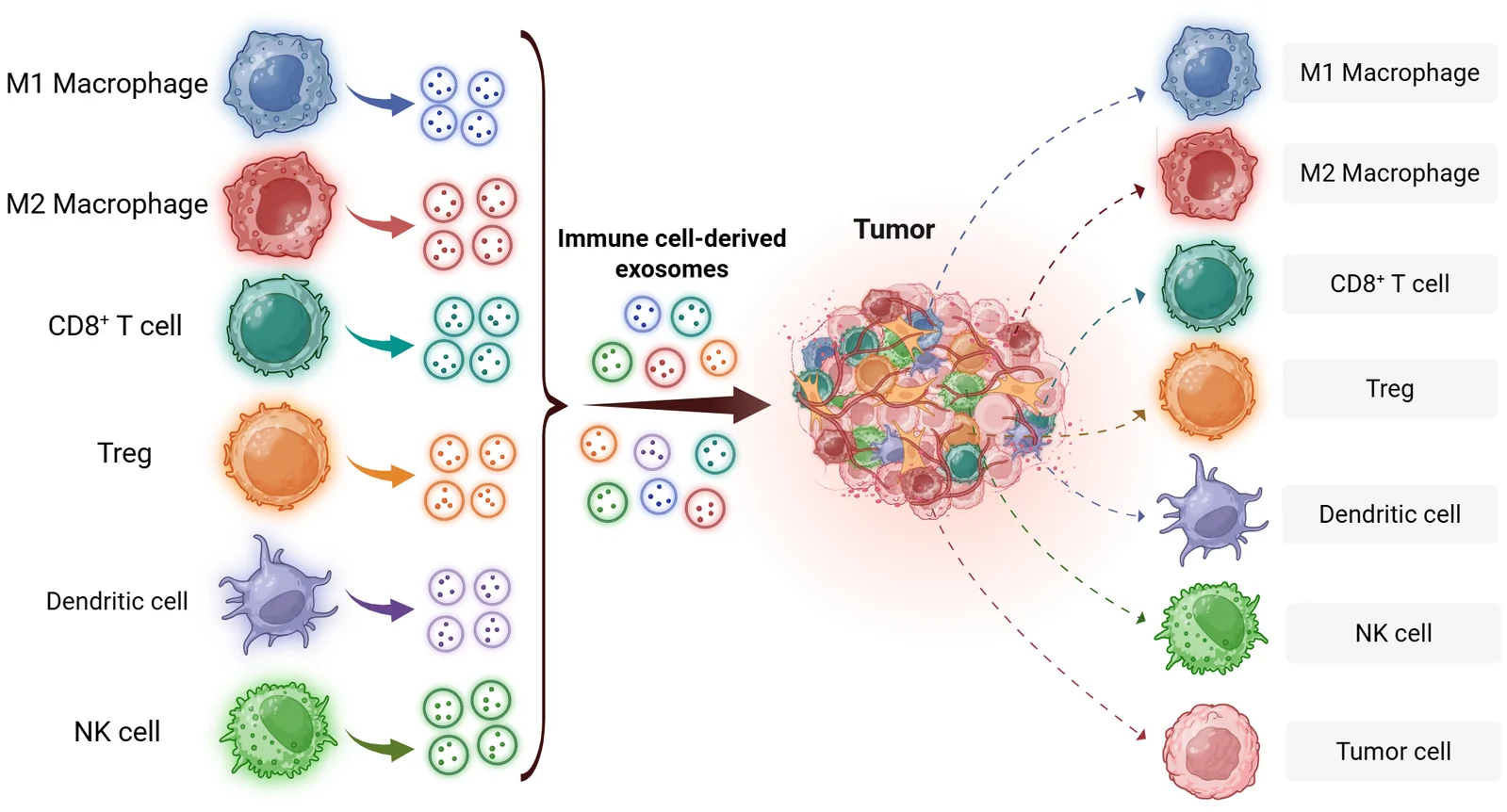
Sources and targets of immune cell-derived exosomes. Exosomes released by M1-like and M2-like macrophages, CD8^+^ T cells, regulatory T cells (Tregs), dendritic cells, and natural killer (NK) cells can mediate communication with macrophages, T-cell subsets, dendritic cells, NK cells, and tumor cells. These interactions may regulate immune activation, immune suppression, macrophage polarization, cytotoxic responses, and tumor-cell response.

Immune cell-derived exosomes act as important messengers between innate and adaptive immune cells and can influence both immune and tumor cell behavior (3, 10). This section briefly introduces the major immune-cell sources of exosomes in the tumor microenvironment before the subsequent mechanism-focused sections. These sources include dendritic cells, natural killer cells, T cells, B cells, macrophages, regulatory T cells, and myeloid-derived suppressor cells (**Figure 2**).

### 2.1 Major immune-cell sources of exosomes

Dendritic cells are antigen-presenting cells that play a role in T-cell activation and immune regulation. Dendritic-derived exosomes can carry immune-related molecules such as major histocompatibility complex (MHC) molecules and co-stimulatory molecules, which can support antigen presentation and improve tumor immunotherapy. They can also increase tumor-cell immunogenicity; in an *in vitro* breast cancer model, tumor cells treated with DC-derived exosomes stimulated greater IFN-γ production by tumor-sensitized T cells, suggesting that these vesicles may make tumor cells more recognizable to immune effector cells (11). In addition, DC-derived exosomes can interact with NK cells and can promote the activation of NK cells. One of their mechanisms is through IL-15Rα and NKG2D surface ligand (12). Overall, dendritic cell-derived exosomes may help connect antigen-presenting cells with effector lymphocytes in the tumor microenvironment. Recent engineering approaches have further expanded the therapeutic potential of dendritic-cell-derived exosomes. Wang et al. developed engineered dendritic-cell-derived exosomes with CCR7-dependent lymph-node targeting, PD-1-mediated checkpoint blockade, and STING-agonist delivery, which remodeled tumor-draining lymph nodes and reduced tumor growth and metastasis in a breast-cancer model (13).

Natural killer cells are important innate immune cells that play a direct role in cytotoxic activity against tumor cells (14). NK-derived exosomes carry cytotoxic and death-related molecules, including FasL, perforin, and granzyme that aid in tumor killing through apoptosis (15, 16). These exosomes have been reported to be effective in lysing myeloid leukemia and melanoma tumors. Surface proteins such as DNAX Accessory Molecule-1 (DNAM1) are expressed on NK-derived exosomes, which support their interaction with tumor cells and contribute to exosome-mediated cytotoxicity (17). In addition to their protein cargo, NK-derived exosomes can also carry miR-186, a tumor suppressor miRNA. In Neuroblastoma, miR-186 targets N-Myc (MYCN) and Aurora Kinase A (AURKA), two genes associated with tumor cell growth and survival. Increasing miR-186 in NK cells also reduces transforming growth factor beta receptor 1 and 2 (TGFBR1 and TGFBR2), which may help prevent TGF-β from suppressing NK cell cytotoxicity (18, 19). Overall, these findings illustrate that NK-cell-derived exosomes can exert antitumor effects through both cytotoxic proteins and regulatory miRNAs. Recent engineering approaches further illustrate the therapeutic potential of NK-cell-derived exosomes. Peng et al. engineered NK-cell-derived exosomes by loading Raddeanin A and modifying their surface with maleimide and mannose. In preclinical models, these engineered exosomes enhanced NK-cell activity and tumor-specific cytotoxic-T-cell-mediated antitumor immunity (20).

T cells are central components of the adaptive immune system and are important in infection, autoimmune diseases, and cancer therapy (15, 21). Exosomes released by cytotoxic T cells (CD8^+^ T cells) can carry cytotoxic molecules, including perforin and granzymes, which may contribute to tumor-cell death (22). However, the effects of T cell-derived exosomes can vary depending on the T-cell subset from which they are released. For example, regulatory T cell-derived exosomes have been shown to suppress CD8^+^ cytotoxic T-cell proliferation and reduce IFN-γ and perforin expression (23). Therefore, T cell-derived exosomes can either support cytotoxic immune responses or promote immune suppression, depending on the type and functional state of the parent T cell. Recent engineering strategies have also enhanced the targeting potential of T-cell-derived exosomes. Engineered human primary CD8^+^ T-cell-derived exosomes displaying IL-2 and an anti-EGFR antibody showed EGFR-dependent targeting and enhanced anticancer activity against A549 lung cancer cells (24).

B cells are components of the adaptive immune system that play a pivotal role in antibody production. Beyond antibody production, B cells also help regulate immune responses in several ways, including presenting antigens, releasing cytokines and providing co-stimulatory signals that support other immune cells (7). Although B cell-derived exosomes remain less explored in cancer immunotherapy, available studies suggest that they have immunomodulatory and potentially immune-stimulating properties. Outside the cancer setting, B cell-derived exosomes carrying allergen peptide-MHC class II complexes promoted antigen-specific CD4^+^ T-cell proliferation and TH2-like cytokine production (25). In contrast, MHC II^+^FasL^+^ exosomes released by EBV-transformed B-lymphoblastoid cells induced apoptosis in antigen-specific CD4^+^ T cells, showing that their effects depend on the cellular source and exosomal cargo (26). In cancer, CD19^+^ B cell-derived extracellular vesicles carrying CD39 and CD73 promoted the conversion of extracellular ATP released after chemotherapy into immunosuppressive adenosine, thereby weakening CD8^+^ T-cell responses. Lower circulating levels of these vesicles were also associated with better outcomes after chemotherapy (27).

Myeloid-Derived Suppressor Cells (MDSC) are immature myeloid cells that can suppress immune responses, especially T-cell activity. Under normal conditions, immature myeloid cells develop into mature immune cells such as granulocytes, macrophages, or dendritic cells (28). However, during cancer, infection, inflammation, or autoimmune diseases, this maturation process can be disrupted, leading to MDSC accumulation (29). Researchers reported that MDSC-derived exosomes can carry immunoregulatory proteins, including members of the S100 family such as S100A8 and S100A9. In colorectal cancer, exosomal S100A9 released by granulocytic MDSCs promoted cancer-cell stemness and tumor growth, indicating that these vesicles can directly support tumor progression (30, 31).

Macrophages are specialized immune cells known as the first line of defense involved in the detection, phagocytosis, and destruction of pathogens and tumor cells (32). In the tumor microenvironment, macrophages can adopt a range of functional states that are described as M1-like and M2-like phenotypes. M1-like macrophages are generally associated with inflammatory and antitumor responses, whereas M2-like macrophages more commonly support immune suppression, tissue remodeling, and tumor progression. However, these states are flexible and can change in response to signals within the tumor microenvironment (33). Macrophage-derived exosomes can reflect the functional state of their parent cells and influence recipient cells through their molecular cargo (34). In colorectal cancer, M2 macrophage-derived exosomes transferred miR-21-5p and miR-155-5p to tumor cells, where they reduced BRG1 expression and promoted tumor-cell migration and invasion (35). Overall, macrophage-derived exosomes can exert different effects depending on the polarization state of the parent macrophage and the cargo carried by the vesicles.

### 2.2 Context-dependent effects of immune-derived exosomes

Immune cell-derived exosomes do not possess fixed antitumor or protumor functions. Instead, their effects depend on several factors including the immune cell that releases them, its activation state, the cargo carried by the vesicles, and the type and condition of the recipient cell. Macrophage-derived exosomes are a clear example of how the polarization state of the parent cell shapes function. Exosomes released by M1-like macrophages are generally associated with inflammatory and antitumor responses, while those that are released by M2-like macrophages or tumor-associated macrophages often carry molecules that support immune suppression, tumor-cell growth, invasion, and metastasis (33–35). Donor-cell activation state exerts a comparable influence on exosome function. Exosomes released by NK cells stimulated with both IL-15 and IL-21 showed stronger cytotoxic and apoptosis-inducing effects against cancer cells than exosomes from unstimulated NK cells. These exosomes increased proapoptotic and cytotoxic proteins, including Bax, cleaved caspase-3, perforin, and granzyme B, while reducing the antiapoptotic protein Bcl-2 (36).

Importantly, the relationship between immune cell-derived exosomes and the tumor microenvironment is reciprocal. Tumor-associated signals can alter the activation state and molecular programs of immune cells, thereby changing the composition and biological activity of the vesicles they subsequently release. In neuroblastoma, NK cells previously exposed to tumor cells released exosomes that enhanced the activation and antitumor activity of recipient NK cells, indicating that tumor-cell exposure can alter the functional properties of NK-cell-derived exosomes (37). Together with the effects of cytokine priming described above, these findings suggest that extracellular signals that are encountered by parent immune cells can shape the biological activity of the vesicles it subsequently releases.

Exosomal cargo composition can also shape biological outcome. In M1 macrophage-derived exosomes, miR-155 and miR-199a contributed to the reprogramming of M2-like macrophages toward an inflammatory phenotype, as inhibition of these miRNAs reduced the reprogramming effect (38). Conversely, ApoE carried by M2 macrophage-derived exosomes contributed to gastric cancer-cell migration, and ApoE-deficient exosomes showed substantially reduced promigratory activity (39). These examples illustrate how specific vesicular cargo can contribute to distinct antitumor or protumor effects.

The status of the recipient cell can redirect the outcome entirely. Although activated CD8^+^ T cells are normally associated with tumor-cell killing, FasL-bearing exosomes from activated CD8^+^ T cells promoted invasion rather than apoptosis in Fas-resistant tumor cells, illustrating how recipient-cell signaling competence can redirect the biological outcome of FasL-mediated signaling (40).

Together, these examples highlight that the biological outcome of immune cell-derived exosomes is shaped by the donor-cell state, exosomal cargo, and signaling characteristics of the recipient tumor cell. This context-dependent behavior provides the rationale for organizing the following sections according to the antitumor and protumor mechanisms regulated by immune cell-derived exosomes.

## 3 Antitumor functions of immune cell-derived exosomes

### 3.1 Antitumor immune activation and cytokine remodeling

Antitumor immune activation in this context refers to exosome-mediated intercellular communication that supports immune recognition and activation of effector lymphocytes involved in tumor control (41–43). Immune cell-derived exosomes can contribute to this process by carrying peptide-MHC complexes, costimulatory or adhesion molecules, activating ligands or receptors, and bioactive protein, cytokine, and RNA cargos that regulate recipient immune-cell responses. Through these cargo-dependent signals, exosomes can promote antigen presentation, CD4^+^ and CD8^+^ T-cell activation, NK-cell activation or proliferation, cytotoxic activity, and antitumor responses (42–44). Furthermore, cytokine remodeling here refers to exosome-associated changes toward an immune-stimulatory effector profile. This includes IFN-γ/TNF-α responses and IL-2-, IL-12-, or IL-15-linked T-cell and NK-cell activity, rather than tolerogenic or immunosuppressive cytokine signaling (42–45).

Consistently, several research studies show these immune-activating functions across different immune cell-derived exosomes. For instance, exosomes isolated from cytokine-activated NK cells previously exposed to neuroblastoma (NB) cells could “educate” recipient NK cells and enhance their antitumor activity. These exosomes expressed natural killer cell receptors, including CD56, NKp30, NKp44, NKp46, NKG2D, and KIR2DL2. Following exosome treatment, recipient NK cells showed increased natural cytotoxicity receptor expression, particularly NKp44, enhanced IFN-γ and TNF-α secretion, and greater cytotoxicity against neuroblastoma cells. Additionally, *in vivo*, pretreatment of NK cells with these exosomes reduced neuroblastoma tumor weight and volume, with maximum effect when combined with cytokine activation. Together, this study supports a model in which NB-exposed NK-cell exosomes enhance NK-cell activation and antitumor function against neuroblastoma (37).

Dendritic cell-derived exosomes provide another route for immune activation by stimulating NK-cell responses. Viaud et al. showed that dendritic cell-derived exosomes promoted IL-15Rα-dependent NK-cell proliferation and NKG2D-dependent NK-cell activation, enhanced NK cytotoxicity, and reduced B16F10 lung metastases in an NK1.1^+^ cell-dependent manner. In human NK-cell assays, these exosomes carried functional IL-15Rα and surface NKG2D ligands, increased CD69 expression, enhanced NK-cell proliferation in the presence of IL-15, and synergized with IL-15 to promote IFN-γ secretion, supporting a role for dendritic cell-derived exosomes in non-MHC-restricted NK-cell antitumor immunity (12).

In addition to NK-cell activation, dendritic cell-derived exosomes can promote antigen-specific adaptive immunity. Näslund et al. compared dendritic cell-derived exosomes loaded with whole OVA protein against exosomes loaded only with SIINFEKL, the OVA-derived CD8^+^ T-cell epitope, in the B16/OVA melanoma model. While both stimulated CD8^+^ T-cell proliferation *in vitro*, whole OVA-loaded dendritic cell-derived exosomes induced robust SIINFEKL-specific CD8^+^ T-cell proliferation and cytotoxic responses *in vivo*, whereas SIINFEKL-loaded exosomes did not induce substantial CD8^+^ T-cell activation *in vivo*. They also found that the response to whole OVA-loaded exosomes was dependent on CD4^+^ T cells and was enhanced by B-cell-mediated mechanisms, and these exosomes provided stronger protective antitumor immunity than peptide-loaded exosomes. The contrasting *in vitro* and *in vivo* responses observed with SIINFEKL-loaded exosomes indicate that direct CD8^+^ T-cell stimulation alone may not predict the *in vivo* immunogenicity of dendritic cell-derived exosomes. Rather, effective antitumor immunity in this model appears to depend on a broader antigenic context that supports CD4^+^ T-cell help and B-cell-mediated contributions to the immune response (46).

In summary, these studies show that immune cell-derived exosomes can amplify antitumor immunity through several mechanisms: educating NK cells, activating NK cells through IL-15Rα/NKG2D-related signaling, and supporting antigen-specific CD8^+^ T-cell responses through CD4^+^ T-cell and B-cell support.

### 3.2 Antitumor macrophage/myeloid reprogramming

Macrophage polarization pathways control the switch between pro-inflammatory M1 and anti-inflammatory M2 states, with a central role in shaping the tumor microenvironment and anti-tumor immunity. While M1 macrophages produce pro-inflammatory cytokines (e.g., IL-12, TNF-α), upregulate MHC II and co-stimulatory molecules, and enhance phagocytosis and CD8^+^ T-cell responses, M2 macrophages produce immunosuppressive mediators that impair antigen presentation and support tumor progression (47–49).

Kim et al. demonstrated that exosomes derived from M1-polarized macrophages (M1-Exo) can directly reprogram tumor-associated macrophages (TAMs) toward an antitumor phenotype. These M1-Exo contained pro-inflammatory miRNAs, including miR-27a, miR-125b, miR-155, and miR-199a. Pathway-enrichment analysis of the M1-exosomal miRNA profile identified predicted target pathways, including endocytosis, proteoglycans in cancer, MAPK signaling, and NF-κB signaling. MAPK and NF-kappaB signaling are known regulators of inflammatory macrophage activation and polarization (50). However, the study did not individually and functionally validate these pathways as mediators of macrophage repolarization. Silencing miR-155 and miR-199a reduced the ability of M1-derived exosomes to convert M2-like TAMs into M1-like macrophages, supporting a functional role for exosomal miRNAs in macrophage reprogramming. Upon M1-Exo uptake, M2-like TAMs showed reduced M2 markers, including CD206, increased M1 markers, including CD40 and CD86, and enhanced phagocytosis, nitric oxide production, and antigen cross-presentation. In 4T1 tumor-bearing mice, intratumoral M1-Exo delayed tumor growth and shifted TAMs toward an M1-like profile. This was translated to a human ex vivo model where M1-Exo decreased CD206/CD163 and increased MHC class II in patient-derived TAMs, further supporting macrophage repolarization toward an antitumor, antigen-presenting phenotype (38).

A related engineered-exosome strategy was reported by Gunassekaran et al., who used IL4R-targeted M1 macrophage-derived exosomes to reprogram M2-like macrophages/TAMs in a 4T1 breast cancer model. Because NF-κB p50 overexpression in TAMs has been linked to suppression of M1-like inflammatory responses and antitumor resistance (51), these exosomes were loaded with NF-κB p50 siRNA and miR-511-3p and modified with IL4RPep-1 to target IL4R-expressing M2-like macrophages/TAMs. Following uptake by M2 macrophages, IL4R-targeted exosomes reduced NF-κB p50 and Rho-associated coiled-coil-containing protein kinase 2 (ROCK2) expression, decreased M2-associated markers such as arginase 1 (Arg1) and IL-10, and increased M1-associated markers including IL-12p40 and iNOS. In 4T1 tumor-bearing mice, this formulation improved tumor homing, reduced tumor growth, decreased immunosuppressive populations including M2 macrophages, IL-10^+^ macrophages, MDSCs, and Tregs, and increased M1 macrophages and CD8^+^ T cells in the tumor microenvironment (52).

Furthermore, a study by Choo et al. demonstrated M1/M2 polarization using exosome-mimetic nanovesicles. Here, they generated M1 macrophage-derived nanovesicles (M1NVs) by serial extrusion of LPS-polarized RAW264.7. These vesicles carried higher levels of M1-associated mRNAs, including CD86, IL-6, TNF-α, and iNOS, as well as polarization-related miRNAs, compared with nanovesicles derived from non-polarized M0 macrophages (M0NVs). M1NVs preferentially entered M2-like macrophages and promoted M2-to-M1 repolarization *in vitro*, increasing M1-associated markers and cytokines such as CD86, IL-6, TNF-α, and iNOS, while reducing M2-associated markers such as IL-4, IL-10, Fizz-1, and CD206. In CT26 tumor-bearing mice, M1NVs enhanced the antitumor effect of anti-PD-L1 therapy. The combination treatment increased M1-associated markers, decreased M2-associated markers, reduced angiogenesis-associated genes such as VEGF and MMP9, and increased CD4^+^ and CD8^+^ T-cell/Treg ratios in the tumor microenvironment. Although this study used exosome-mimetic nanovesicles, it supports the broader concept that macrophage-derived vesicles can remodel TAM polarization and improve antitumor immune responses (53).

Collectively, these studies support the ability of macrophage-derived vesicles to reprogram TAMs toward a more inflammatory and T-cell-supportive phenotype, although the strength and physiological relevance of the mechanistic evidence differ across models. In natural M1-derived exosomes, miR-155 and miR-199a contributed to macrophage reprogramming, but their inhibition did not completely reverse the effect, indicating involvement of additional factors (38). The engineered exosome study using IL4R-targeted M1 exosomes provides more controlled evidence for defined cargo effects through defined cargo loading and receptor-directed delivery, although this engineered system may not fully represent endogenous exosomal signaling (52). Exosome-mimetic nanovesicles extend this concept, but their artificial production limits direct extrapolation to naturally secreted exosomes (53). Overall, the findings are promising, although most *in vivo* evidence remains derived from murine tumor models.

### 3.3 Checkpoint reversal and restoration of effector immunity

Programmed death-ligand 1 (PD-L1) and other immune-checkpoint molecules form a regulatory module that limits T-cell activation and effector function, allowing tumors to escape immune surveillance (54–57). Within the tumor microenvironment, checkpoint ligands expressed by tumor cells, immune cells, or carried by extracellular vesicles can engage inhibitory receptors on effector lymphocytes and limit T-cell proliferation, cytokine production, and cytotoxic activity. Specifically, binding of PD-L1 to PD-1 on activated T cells delivers inhibitory signals, and high or sustained PD-L1 expression is associated with poor clinical outcomes and resistance to immunotherapy in several cancers (57–60). Although checkpoint inhibitors have transformed cancer treatment, variable and often incomplete responses highlight the need to understand upstream mechanisms that regulate checkpoint signaling (61, 62). In this context, checkpoint reversal refers to the ability of immune cell-derived exosomes to attenuate PD-1/PD-L1, CTLA-4, or related inhibitory pathways and thereby restore effector-cell proliferation, cytokine secretion, cytotoxic molecule expression, and tumor-cell sensitivity to immune-mediated killing (63, 64).

PI3K-AKT-mTOR signaling is a major tumor-intrinsic pathway that regulates cancer cell growth, proliferation, metabolism, and survival (65–67). In addition to these tumor-promoting functions, aberrant PI3K-AKT-mTOR activation has been associated with increased PD-L1 expression through transcriptional, post-transcriptional, and translational mechanisms reported across different tumor contexts (62, 68). A study by Xie et al. showed that liver cancer cells, particularly HepG2 cells, treated with NK-cell-derived exosomes (NK-exo) showed reduced cell viability, migration, and invasion, increased apoptosis, and lower PD-L1 expression on the tumor cell surface. When NK-exo-treated HepG2 cells were co-cultured with CD8^+^ T cells, they enhanced CD8^+^ T-cell proliferation and increased secretion of IL-2, granzyme B, and TNF-α. Mechanistically, NK-exo suppressed PI3K/AKT/mTOR signaling in HepG2 cells. Levels of p-PI3K, p-AKT, p-mTOR, and PD-L1 decreased after NK-exo treatment, and these changes were restored by Recilisib, a PI3K agonist, indicating that NK-exo downregulates PD-L1 through inhibition of the PI3K/AKT/mTOR pathway. Moreover, in an H22 liver cancer mouse model, NK-exo similarly reduced tumor PD-L1 and p-PI3K/p-AKT/p-mTOR, increased intratumoral CD8^+^ T-cell infiltration and effector cytokines, and inhibited tumor growth. These findings suggest that NK-exo enhance CD8^+^ T-cell-mediated antitumor immunity in liver cancer by repressing PI3K/AKT/mTOR signaling in tumor cells and lowering PD-L1 expression on their surface. However, the specific molecular intermediates linking PI3K-AKT-mTOR inhibition to reduced PD-L1 expression were not identified in this model (69).

Jung et al. developed IL-2-tethered small extracellular vesicles (IL2-sEVs) from engineered Jurkat T cells expressing membrane-bound IL-2 via a flexible linker. IL2-sEVs increased CD8^+^ T-cell proliferation and activation, upregulated effector molecules such as IFN-γ, perforin, and granzyme B, and reduced inhibitory receptors PD-1 and CTLA-4, without expanding CD4^+^CD25^+^ Treg cells. In melanoma cells, IL2-sEVs downregulated both cellular and exosomal PD-L1, partly through IL2-sEV-enriched miRNAs including miR-181a-3p and miR-223-3p, which reduced PD-L1 protein levels. IL-2-sEVs also reduced Rab27a and other Rab GTPases involved in sEV secretion. Additionally, in syngeneic B16 melanoma models, IL2-sEVs inhibited primary tumor growth more effectively than non-engineered sEVs or recombinant IL-2. This led to reduced lung metastasis compared with control sEVs, decreased tumor and plasma ePD-L1, and improved the efficacy of cisplatin and anti-PD-L1 antibody (63).

Taken together, these studies provide complementary evidence for checkpoint modulation by immune cell-derived vesicles. The NK-exosome study provided pathway-level evidence because reactivation of PI3K signaling reversed PD-L1 downregulation and associated CD8^+^ T-cell responses, although the responsible exosomal cargo was not identified (69). In contrast, the IL2-sEV study used an engineered vesicle platform which may not fully reflect endogenous T-cell-derived sEV signaling (63).

### 3.4 Cytotoxic and cell-death signaling

Apoptosis is a programmed cell-death pathway that eliminates damaged or malignant cells through coordinated activation of pro-apoptotic proteins and caspases (70, 71). In cytotoxic lymphocyte-mediated tumor killing, apoptosis can be triggered through intrinsic mitochondrial and extrinsic death-receptor pathways (72, 73). The intrinsic pathway is regulated in part by the balance between pro-apoptotic proteins such as Bax and anti-apoptotic proteins such as Bcl-2, leading to mitochondrial cytochrome c release, caspase-9 activation, and downstream activation of executioner caspases such as caspase-3 and caspase-7. The extrinsic pathway is initiated by death ligands such as FasL and TRAIL, which activate caspase-8 through death-receptor signaling. Both intrinsic and extrinsic pathways converge on executioner caspase activation, poly(ADP-ribose) polymerase (PARP) cleavage, and apoptotic cell death (70, 71, 73). Cytotoxic immune mediators, including perforin and granzyme B, can further engage apoptotic machinery by delivering granzyme B into target cells and promoting caspase-dependent tumor-cell death (73, 74).

In tumor cells, sensitivity to apoptosis is also shaped by survival pathways such as PI3K-AKT and MAPK-ERK, which promote proliferation and resistance to cell death when activated (75, 76). In hepatocellular carcinoma (HCC), Kim et al. showed that human NK-92 cell-derived exosomes suppress tumor growth through two linked mechanisms: apoptosis induction and inhibition of survival signaling. NK-exosomes were internalized by HCC cells and carried cytotoxic/death-ligand proteins, including perforin, granzyme B, FasL, and TRAIL. At the mechanistic level, NK-exosomes reduced AKT and ERK1/2 phosphorylation, suggesting inhibition of PI3K-AKT and MAPK-ERK survival/proliferation signaling. In NK-exosome-treated cells, the detectable levels of perforin and granzyme B increased. Cytochrome c and cleaved caspase-9 also increased, supporting mitochondrial apoptotic signaling. In parallel, increased FasL, TRAIL, and cleaved caspase-8 supported death-receptor signaling. Downstream, NK-exosome treatment increased cleaved caspase-3, cleaved caspase-7, and cleaved PARP, further supporting caspase-dependent apoptotic cell death. Importantly, NK-exosomes showed tumor-homing ability in a subcutaneous HCC model and reduced tumor burden in an orthotopic HCC model. Treated tumors also showed increased apoptosis-associated signaling, including higher cleaved PARP and cleaved caspase levels (77).

A related *in vitro* study from the same group showed that cytokine priming can strengthen the cytotoxic activity of NK-exosomes against HCC cells. After IL-15/IL-21 stimulation, NK-92 cell-derived exosomes showed increased perforin and granzyme B levels without a major change in uptake by Hep3B cells. Compared with non-primed NK-exosomes, IL-15/IL-21-primed NK-exosomes more strongly reduced Hep3B cell viability and increased apoptotic cell death. Mechanistically, this enhanced effect was associated with increased pro-apoptotic signaling and reduced anti-apoptotic Bcl-2 expression. These findings suggest that IL-15/IL-21 priming can further enhance the perforin/granzyme B-associated apoptotic activity of NK-exosomes in HCC cells (36).

Wu et al. expanded this cytotoxicity framework by showing that activated human NK-cell-derived EVs can kill cancer cells through multiple, cell-death mechanisms rather than a single apoptotic mechanism. The levels of cytotoxic proteins in NK-EVs, including perforin, granzyme A, granzyme B, and granulysin, showed moderate positive correlations with cytotoxicity against CHLA255 neuroblastoma and SupB15 acute lymphoblastic leukemia cells. Mechanistically, NK-EVs reduced the granzyme A substrates SET and HMG2, supporting a caspase-independent death pathway. They also induced mitochondrial cytochrome c release and altered ER-stress-associated markers, suggesting additional caspase-dependent and ER-stress-associated mechanisms (16).

In another study by Shi et al. sEVs from IL-12/IL-15/IL-18-induced memory-like NK cells (mNK-sEV) showed stronger cytotoxic and antitumor activity than sEVs from conventionally cultured NK cells (conNK-sEV). mNK-sEV entered MGC803 gastric cancer and A549 lung cancer cells through macropinocytosis and induced caspase-dependent apoptosis, supported by increased cytochrome c, cleaved caspase-8, cleaved caspase-3, and cleaved PARP, as well as reduced apoptosis after zVAD-fmk treatment. In the MGC803 xenograft model, mNK-sEV inhibited tumor growth more effectively than conNK-sEV and showed higher tumor accumulation. This enhanced activity was partly linked to higher granulysin content, because treatment with a granulysin-neutralizing monoclonal antibody reduced mNK-sEV-induced tumor-cell apoptosis and decreased apoptosis-associated signaling (78).

Di Pace et al. showed that DNAM1 on human NK-cell-derived exosomes contributes to exosome-mediated tumor-cell cytotoxicity. NK exosomes induced dose-dependent cytotoxicity against K562 erythroleukemia and NALM-18 B-acute lymphoblastic leukemia cells. In NALM-18 cells, blocking the DNAM1 ligands CD155 and CD112 reduced NK-exosome-mediated cytotoxicity and delayed caspase-3/7-associated apoptosis. Blocking DNAM1 on the exosome surface also reduced cytotoxicity, supporting a role for DNAM1 binding to its ligands in NK-exosome-mediated tumor-cell killing (17).

Collectively, these studies indicate that NK-cell-derived vesicles can trigger tumor-cell death through multiple mechanisms. However, the strength of evidence for individual mechanisms varies across studies. Caspase-dependent apoptosis, granulysin, and DNAM1 were supported by inhibitor or blocking experiments, whereas the roles of several other cytotoxic proteins and signaling pathways were mainly inferred from changes in protein levels, apoptosis-related markers, or correlations with cytotoxicity (16, 17, 36, 77, 78). These findings suggest that the dominant death mechanism may vary depending on the activation state of the NK cells, vesicle composition, and the recipient tumor-cell type.

### 3.5 Inhibition of tumor progression and metastasis

Immune cell-derived exosomes can also restrain tumor progression by transferring regulatory cargo that interferes with tumor-intrinsic pathways. In this way, they can alter proliferation, epithelial-mesenchymal transition (EMT), invasion, migration, tumor growth, and metastasis-associated signaling (79). In several studies, researchers focused on immune exosomes that suppress cancer progression programs by regulating oncogenic, metastasis-associated, or stromal pathways. Zhou et al. showed that CD45RO^-^CD8^+^ T cell-derived exosomes restrict estrogen-driven uterine corpus endometrial cancer (UCEC) progression. This effect involved ERβ/miR-765/PLP2/Notch axis. In UCEC cells, estrogen reduced miR-765 and increased PLP2, partly through estrogen receptor beta (ERβ)-related signaling. PLP2 activated Notch signaling, which promoted proliferation, EMT, invasion, and tumor progression. Importantly, CD45RO^-^CD8^+^ T cell-derived exosomes were enriched in miR-765. These exosomes increased miR-765 in UCEC cells, reduced PLP2 expression, and partly reversed estrogen-induced proliferation, EMT, invasion, and tumor growth. Together, this study suggests that CD45RO^-^CD8^+^ T cell-derived exosomal miR-765 counteracts estrogen-driven UCEC progression by suppressing the PLP2/Notch progression pathway (80).

Another study demonstrated that immune cell-derived exosomal miRNA can suppress tumor progression by targeting the tumor-promoting signaling pathway. Neviani et al. identified miR-186 as a functional cargo of NK cell-derived exosomes in neuroblastoma. NK exosomes carried miR-186 and delivered RNA cargo to MYCN-amplified neuroblastoma cells. These exosomes showed cytotoxic activity against neuroblastoma cells, and this activity was reduced when miR-186 was downregulated in NK cells and their derived exosomes. This suggests that miR-186 partly contributes to NK-exosome-mediated cytotoxicity. Mechanistically, miR-186 directly targeted MYCN, AURKA, TGFBR1, and TGFBR2. This reduced MYCN/AURKA expression and weakened TGF-β pathway activity. In MYCN-amplified neuroblastoma cells, miR-186 reduced tumor-cell growth, migration, and EMT-associated gene expression. In NK cells, targeted miR-186 delivery reduced TGF-β pathway components and prevented TGF-β from suppressing NK-cell cytotoxicity. *In vivo*, delivery of miR-186 through anti-GD2 nanoparticles reduced neuroblastoma tumor burden and improved survival. Collectively, this study suggests that NK-exosomal miR-186 can limit neuroblastoma growth and TGF-β-dependent immune escape (18).

Seo et al. identified another mechanism by which activated CD8^+^ T-cell-derived EVs can limit tumor progression by targeting mesenchymal cells within the tumor stroma. These EVs were preferentially taken up by CD140a^+^Sca-1^+^ mesenchymal stromal cells and induced caspase-3-dependent apoptosis. The EVs were enriched in miR-298-5p, which contributed to this effect, although its direct target was not identified. Treatment reduced mesenchymal stem cell and cancer-associated fibroblast areas within tumors and lowered CD140a and TGF-β expression. In B16F10, 4T1, and CMS5m tumor models, treatment reduced local invasion and, in B16F10 and CMS5m models, decreased lung metastasis. Together, this study suggests that activated CD8^+^ T-cell-derived EVs can limit invasion and metastasis by disrupting tumor-supportive mesenchymal stroma (81).

**Figure 3:**
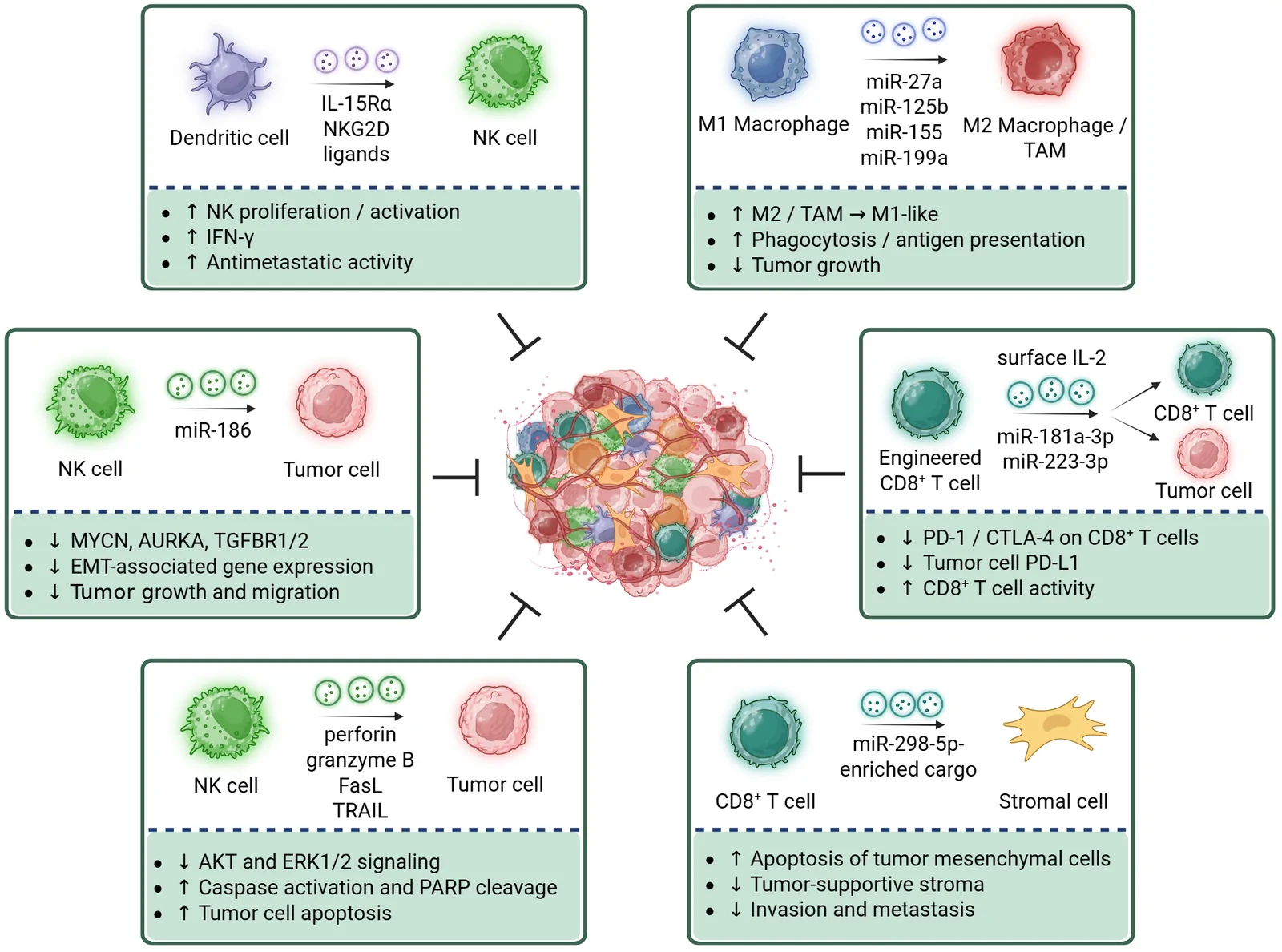
Representative antitumor mechanisms of immune cell-derived exosomes. Immune cell-derived exosome can activate NK and CD8^+^ T cells, reprogram tumor-associated macrophages toward M1 state, suppress immune-checkpoint signaling, induce apoptotic tumor-cell death, suppress tumor-promoting or metastatic pathways, and disrupt tumor-supportive stromal cells. Through these mechanisms, they can reduce tumor growth, invasion, immune escape, and metastasis.

In summary, these studies show that immune cell-derived exosomes and EVs can restrain tumor progression by suppressing tumor-cell oncogenic programs, counteracting immune escape, and disrupting stromal mechanisms that support invasion and metastasis (**Figure 3**). The summaries of studies in section 3 are provided in **Table 1**.

**Table 1:** Antitumor mechanisms of immune cell-derived exosomes in cancer.

Antitumor mechanism	Immune–cell source and vesicle type	Key cargo or feature	Primary recipient/target cell	Main target/pathway	Main antitumor effect	Cancer type	Ref.
Immune activation and cytokine remodeling	Exosomes from cytokine–activated NK cells previously exposed to neuroblastoma cells	NK–cell receptors, including CD56, NKp30, NKp44, NKp46, NKG2D, and KIR2DL2	Recipient NK cells	↑ Natural cytotoxicity receptor expression, particularly NKp44	↑ IFN–γ and TNF–α secretion and NK–cell cytotoxicity, ↓ tumor growth	Neuroblastoma	(37)
	DC–derived exosomes	IL–15Rα and NKG2D ligands	NK cells	IL–15Rα– and NKG2D–mediated signaling	↑ NK–cell proliferation, ↑ IFN–γ production, and cytotoxicity, ↓ lung metastasis	Melanoma	(12)
	DC–derived exosomes	Whole OVA protein or SIINFEKL peptide	CD8^+^ T cells, with CD4^+^ T–cell and B–cell support	Antigen presentation and antigen–specific T–cell priming	↑ CD8^+^ T–cell proliferation, cytotoxicity, and protective immunity, particularly with whole OVA–loaded exosomes	Melanoma	(46)
Macrophage/myeloid reprogramming	M1 macrophage–derived exosomes	miR–27a, miR–125b, miR–155, and miR–199a	M2–like macrophages and TAMs	miR–155/miR–199a–dependent M2→M1 reprogramming, NF–κB/MAPK–associated programs	↓ M2 markers, ↑ M1 markers, ↑ antigen presentation, ↓ tumor growth	Breast cancer	(38)
	IL4R–targeted M1 macrophage–derived exosomes	NF–κB p50 siRNA, miR–511–3p, and IL4RPep–1	IL4R^+^ M2–like macrophages and TAMs	↓ NF–κB p50 and ROCK2	↑ M1–like macrophages and CD8^+^ T cells, ↓ M2–associated macrophages, MDSCs, Tregs, and tumor growth	Breast cancer	(52)
	M1 macrophage–derived exosome–mimetic nanovesicles	M1–associated mRNAs and miRNAs	M2–like macrophages	M2→M1 reprogramming	↑ M1–associated responses and T–cell/Treg ratios, ↓ M2– and angiogenesis–associated programs, ↑ anti–PD–L1 efficacy	Colorectal cancer	(53)
Checkpoint reversal and restoration of effector immunity	NK–cell–derived exosomes	Specific cargo not identified	Liver cancer cells, indirectly CD8^+^ T cells	↓ PI3K–AKT–mTOR signaling and tumor–cell PD–L1	↓ Tumor–cell viability, migration, invasion, and tumor growth, ↑ apoptosis and CD8^+^ T–cell responses	Liver cancer	(69)
	IL–2–tethered sEVs from engineered Jurkat T cells	Surface IL–2, miR–181a–3p, and miR–223–3p	CD8^+^ T cells and melanoma cells	↓ PD–1 and CTLA–4 on T cells, ↓ cellular and exosomal PD–L1 and Rab GTPases	↑ CD8^+^ T–cell proliferation, effector function, and cytotoxicity, ↓ tumor growth and metastasis	Melanoma	(63)
Cytotoxic and cell–death signaling	NK–92 cell–derived exosomes	Perforin, granzyme B, FasL, and TRAIL	HCC cells	↓ AKT and ERK1/2 signaling, activation of mitochondrial and death–receptor apoptotic pathways, ↑ Caspase activation and PARP cleavage	↑ Tumor–cell apoptosis, ↓ tumor burden	Hepatocellular carcinoma	(77)
	IL–15/IL–21–primed NK–92 cell–derived exosomes	Perforin and granzyme B	HCC cells	↑ Bax, cleaved caspase–3, and cleaved PARP, ↓ Bcl–2	↑ Apoptosis, ↓ tumor–cell viability	Hepatocellular carcinoma	(36)
	Activated human NK–cell–derived EVs	Perforin, granzyme A, granzyme B, and granulysin	Neuroblastoma and leukemia cells	↓ SET/HMG2, ↑ cytochrome c release and ER–stress signaling, caspase–dependent and –independent cell death	↑ Tumor–cell death and cytotoxicity	Neuroblastoma and B–cell acute lymphoblastic leukemia	(16)
	Memory–like NK–cell sEVs (IL–12/IL–15/IL–18–primed)	Granulysin	Gastric and lung cancer cells	↑ Cytochrome c, cleaved caspase–8, cleaved caspase–3, and cleaved PARP	↑ Tumor–cell Apoptosis, ↓ tumor growth	Gastric and lung cancer	(78)
	Human NK–cell–derived exosomes	Surface DNAM1	CD155^+^/CD112^+^ leukemia cells	DNAM1CD155/CD112 interaction, ↑ caspase–3/7 activation	↑ Tumor–cell killing and apoptosis	Erythroleukemia and B–cell acute lymphoblastic leukemia	(17)
Inhibition of tumor progression and metastasis	CD45RO^-^CD8^+^ T–cell–derived exosomes	miR–765	UCEC cells	↓ PLP2 and Notch signaling through the ERβ/miR–765/PLP2 axis	↓ Proliferation, EMT, invasion, and tumor growth	Uterine corpus endometrial cancer	(80)
	NK–cell–derived exosomes	miR–186	Neuroblastoma cells	↓ MYCN, AURKA, TGFBR1, and TGFBR2	↓ Neuroblastoma–cell growth, migration, and EMT–associated gene expression	Neuroblastoma	(18)
	Activated CD8^+^ T–cell–derived EVs	miR–298–5p	CD140a^+^Sca–1^+^ mesenchymal stromal cells	↑ Caspase–3–dependent apoptosis, ↓ CD140a and TGF–β expression	↓ Tumor–supportive stromal and CAF areas, local invasion, and lung metastasis	Melanoma, breast cancer, and sarcoma	(81)

↑ indicates increased/upregulated expression or activity.

↓ indicates decreased/downregulated expression or activity.

## 4 Protumor and immunosuppressive functions of immune cell-derived exosomes

### 4.1 Immune suppression, tolerogenic reprogramming, and cytokine-linked immune escape

Immune cell-derived exosomes can also promote tumor immune escape by shifting immune communication toward suppressive or tolerogenic responses. Through their surface molecules and regulatory cargo, these vesicles can reduce immune activation, weaken effector-cell function, and support conditions that favor tumor persistence (8). In this section, we discuss studies showing how immune-cell-derived exosomes contribute to immunosuppression through effects on antigen-presenting cells, T-cell responses, and cytokine-linked immune regulation.

Tung et al. showed that Treg-derived EVs can transfer miRNAs to dendritic cells and shift their cytokine response toward a tolerogenic profile. Specifically, CD4^+^CD25^+^FoxP3^+^ Treg-derived EVs increased miR-150-5p and miR-142-3p levels in dendritic cells and altered LPS-induced cytokine production, increasing IL-10 and reducing IL-6 without markedly affecting TNF-α or CD80 (82). Furthermore, a related study by Smyth et al. showed that CD4^+^CD25^+^Foxp3^+^ Treg-derived exosomes can suppress CD4^+^ T-cell responses through a surface-enzyme mechanism. Treg-derived exosomes expressed CD73, enabling the conversion of extracellular AMP to adenosine. This enzymatic activity was associated with reduced CD4^+^ T-cell proliferation and lower IL-2 and IFN-γ production *in vitro*. The suppressive effect was not mediated by CTLA-4, and CD73-negative CD4^+^ T-cell exosomes did not show similar regulatory activity. These results identify exosomal CD73 as a surface-enzyme mediator of Treg-exosome-mediated immune suppression (83). Although these two studies were not conducted in cancer models, they demonstrate Treg-derived exosome-mediated mechanisms that may contribute to immunosuppression in the tumor microenvironment. Consistent with these suppressive roles, Xie et al. showed that exosomes from natural CD8^+^CD25^+^ regulatory T cells suppress DC-induced CD8^+^ T-cell expansion, CTL activity, and protective antitumor immunity in a melanoma model (84).

Overall, these studies show that regulatory T-cell-derived vesicles can suppress immune activation at multiple levels, including dendritic-cell cytokine programming, CD4^+^ T-cell proliferation and cytokine production, and CTL-mediated antitumor immunity.

Beyond regulatory T-cell-derived vesicles, macrophage-derived exosomes can also promote tumor immune escape by reshaping CD4^+^ T-cell differentiation and cytokine-linked tumor-immune interactions. The transcription factor STAT3 is an important regulator of CD4^+^ T-cell differentiation and is particularly involved in maintaining the balance between Th17 cells and Tregs. Disruption of this axis can favor a more suppressive CD4^+^ T-cell profile in the tumor microenvironment (85, 86). Zhou et al. showed that M2/TAM-derived exosomes taken up by CD4^+^ T cells increased the Treg/Th17 ratio in epithelial ovarian cancer (EOC). These exosomes were enriched in miR-21-5p and miR-29a-3p, which directly targeted STAT3 and reduced STAT3 expression in CD4^+^ T-cell subsets (87).

Consistent with the role of STAT3 in CD4^+^ T-cell differentiation (85, 86), these miRNAs were associated with a shift toward a higher Treg/Th17 ratio. This shift was accompanied by increased IL-10 and TGF-β and reduced IL-6 and TNF-α. *In vivo*, CD4^+^ T cells transfected with miR-21-5p and miR-29a-3p promoted ovarian tumor growth and metastasis. The findings from this study suggests that the interaction between TAM-derived exosomes and CD4^+^ T-cells generates an immunosuppressive microenvironment that supports tumor progression and metastasis (87).

Beyond reshaping T-cell differentiation, M2 macrophage-derived exosomes can also promote immune escape by changing cytokine production in tumor cells. IL-6 is a tumor-promoting inflammatory cytokine that weakens T-cell-mediated antitumor immunity (88), and its expression is partly regulated by RNA-binding proteins such as ZC3H12B/MCPIP2. Ma et al. showed that M2 macrophage-derived exosomes promote immune escape in colon cancer through a miR-155-5p/ZC3H12B/IL-6 axis. These exosomes were enriched in miR-155-5p and transferred this miRNA to colon cancer cells, where it directly targeted ZC3H12B and reduced its expression (89). Because ZC3H12B normally promotes IL-6 mRNA degradation, its suppression increased IL-6 expression and secretion (89, 90). Subsequently, the axis enhanced colon cancer cell proliferation and resistance to apoptosis while reducing CD3^+^ T-cell proliferation and IFN-γ^+^ T-cell responses. Collectively, these findings suggest that M2 macrophage-derived exosomal miR-155-5p promotes colon cancer immune escape by disrupting ZC3H12B-mediated control of IL-6 and strengthening IL-6-linked tumor-immune suppression (89).

In summary, immune-cell-derived exosomes can suppress antitumor immunity through several routes, including modulation of dendritic-cell function, direct suppression of T-cell responses, altered CD4^+^ T-cell differentiation, and cytokine-linked tumor-immune signaling (82–84, 87, 89). Importantly, the Treg-EV studies by Tung et al. and Smyth et al. were conducted outside cancer models and should therefore be interpreted as mechanistic context rather than direct evidence of tumor-associated immunosuppression (82, 83).

### 4.2 Checkpoint-linked immune escape

In contrast to the immune cell-derived exosomes discussed in Section 3.3, which attenuate PD-1/PD-L1, CTLA-4, or related inhibitory checkpoint pathways, the exosomes discussed here promote tumor immune escape by enhancing checkpoint signaling in tumor cells. Wang et al. showed that M2 macrophage-derived exosomes promote gastric cancer progression, and this was associated with increased PD-L1 expression. Following uptake of M2 exosomes by gastric cancer cells, tumor cell proliferation and migration increased, while apoptosis decreased. From a mechanistic standpoint, these exosomes increased p38 MAPK phosphorylation and PD-L1 protein expression, while other tested pathways, including PTEN/AKT and Ras-ERK1/2, were not significantly changed. Overall, it suggests that M2 macrophage-derived exosomes may support gastric cancer progression and checkpoint-linked immune escape through p38 MAPK-associated PD-L1 upregulation (91).

Weng et al. also provided strong evidence that TAM-derived exosomes promote checkpoint-linked immune escape. In cervical cancer, exosomes derived from M2-polarized TAMs upregulated both PD-L1 mRNA and protein expression in tumor cells without reducing cell viability. This led to these tumor cells becoming more resistant to antigen-specific CD8^+^ T-cell-mediated killing. In addition, IFN-γ, CXCL9, and CXCL10 levels were also reduced within co-cultures. Most importantly, PD-L1 knockdown reduced the suppressive effect of TAM-derived exosomes, showing that this effect depends on tumor-cell PD-L1. *In vivo* studies supported these findings, as administration of these exosomes promoted TC-1 tumor growth, increased tumor PD-L1 expression, reduced CD8^+^ T-cell infiltration, and weakened the response to anti-PD-1 therapy (92).

Collectively, these studies support M2/TAM-derived exosomes as mediators of checkpoint-linked immune escape, although the strength of mechanistic evidence differs. In the gastric cancer study, p38 MAPK activation and PD-L1 upregulation occurred in parallel, but their relationship was not directly confirmed by pathway inhibition. In contrast, the cervical cancer study demonstrated that exosome-mediated immune suppression was functionally dependent on tumor-cell PD-L1 through genetic knockdown and checkpoint blockade. Notably, neither study identified the specific exosomal cargo responsible for PD-L1 upregulation (91, 92).

### 4.3 Tumor-promoting signaling, metabolic reprogramming, and metastatic progression

Immune-derived exosomes can actively promote tumor progression by transferring molecular cargo that rewires tumor-cell-intrinsic programs supporting growth, invasion, metastasis, and therapy resistance. PI3K-AKT signaling is broadly implicated in cytoskeletal regulation, EMT, and metastatic progression in gastric cancer (39, 93, 94). In a study by Zheng et al., M2 macrophage-derived exosomes have been shown to promote tumor-cell migration and invasion through exosomal ApoE. Upon uptake by gastric cancer cells, exosomal ApoE activated the PI3K-AKT signaling, including downstream mTOR phosphorylation (39). This was accompanied by increased expression of EMT-associated and invasion-related proteins, actin cytoskeletal remodeling, and enhanced tumor-cell motility. Exosomes from Apoe-deficient M2 macrophages failed to activate PI3K-AKT signaling or induce the associated cytoskeletal and EMT-related changes, supporting ApoE as a functional mediator of this phenotype (39).

Macrophage-derived exosomes can converge on PI3K-AKT-mTOR signaling through distinct upstream cargos. In another study by Fang et al., M2 macrophage-derived exosomal miR-155-5p promoted non-small-cell lung-cancer proliferation, migration, epithelial-mesenchymal transition, and tumorigenicity. Mechanistically, exosomal miR-155-5p interacted with HuR, increased VEGFR2 stability and expression, and activated the VEGFR2/PI3K/AKT/mTOR axis; VEGFR2 knockdown and pharmacological PI3K inhibition attenuated these effects (95).

Similarly, mast cell-derived exosomes provide another example immune cell-derived vesicles that can activate tumor-promoting signaling in cancer cells. According to Xiao et al., HMC-1 mast cell-derived exosomes enhanced the proliferation and migration of A549 lung adenocarcinoma cells. Mechanistically, these exosomes transferred proto-oncogene receptor tyrosine kinase (KIT) protein to A549 cells. Because A549 cells expressed stem cell factor (SCF), exosomal KIT transfer was associated with activation of KIT-SCF-linked signaling. Consistent with this, mast cell-derived exosomes increased phosphorylation of PI3K, AKT, and GSK3β, increased cyclin D1 expression, and enhanced tumor-cell proliferation. However, although KIT transfer and downstream PI3K-AKT-GSK3β signaling were observed, selective KIT depletion or inhibition was not used to establish that exosomal KIT was required for the proliferative or migratory effects (96).

In a separate gastric-cancer study, Wang et al. also found that M2 tumor-associated macrophage-derived exosomes promoted gastric cancer progression by transferring the lncRNA MALAT1 (metastasis-associated lung adenocarcinoma transcript 1) to gastric cancer cells. Exosomal MALAT1 enhanced aerobic glycolysis, and this metabolic shift was associated with greater tumor-cell proliferation, migration, invasion, metastasis, and resistance to platinum-based chemotherapy. Mechanistically, MALAT1 acted through two related signaling routes: it stabilized δ-catenin by suppressing its ubiquitination and degradation, which in turn supported β-catenin signaling, and it also increased HIF-1α expression by functioning as a sponge for miR-217-5p. Through these mechanisms, MALAT1 activated β-catenin and HIF-1α signaling and promoted glycolytic reprogramming. *In vivo* findings demonstrated that these exosomes also promoted gastric tumor growth, increased metastatic nodules in liver and colon tissues, and reduced the therapeutic effect of oxaliplatin (97).

Furthermore, death-receptor signaling can also be redirected toward tumor-promoting outcomes. Although Fas/FasL signaling classically induces apoptosis in sensitive cells, Fas-resistant tumor cells can convert this pathway into pro-invasive signaling (40, 98, 99). Interestingly, Cai et al. showed that activated CD8^+^ T-cell-derived exosomes carrying FasL did not increase apoptosis or proliferation in melanoma and lung carcinoma cells, but they enhanced their invasive behavior. Mechanistically, exosomal FasL engaged tumor-cell Fas and increased c-FLIP(L), shifting downstream signaling toward ERK and NF-κB activation. These pathways increased MMP9 expression and promoted tumor-cell invasion. *In vivo*, these FasL^+^ T-cell exosomes facilitated metastatic spread of B16 melanoma cells to the lungs, and this effect was reduced when FasL was neutralized. Thus, T-cell-derived exosomes can paradoxically promote tumor progression by redirecting Fas signaling from apoptosis toward a c-FLIP(L)/ERK/NF-κB/MMP9 pro-invasive program (40).

**Figure 4:**
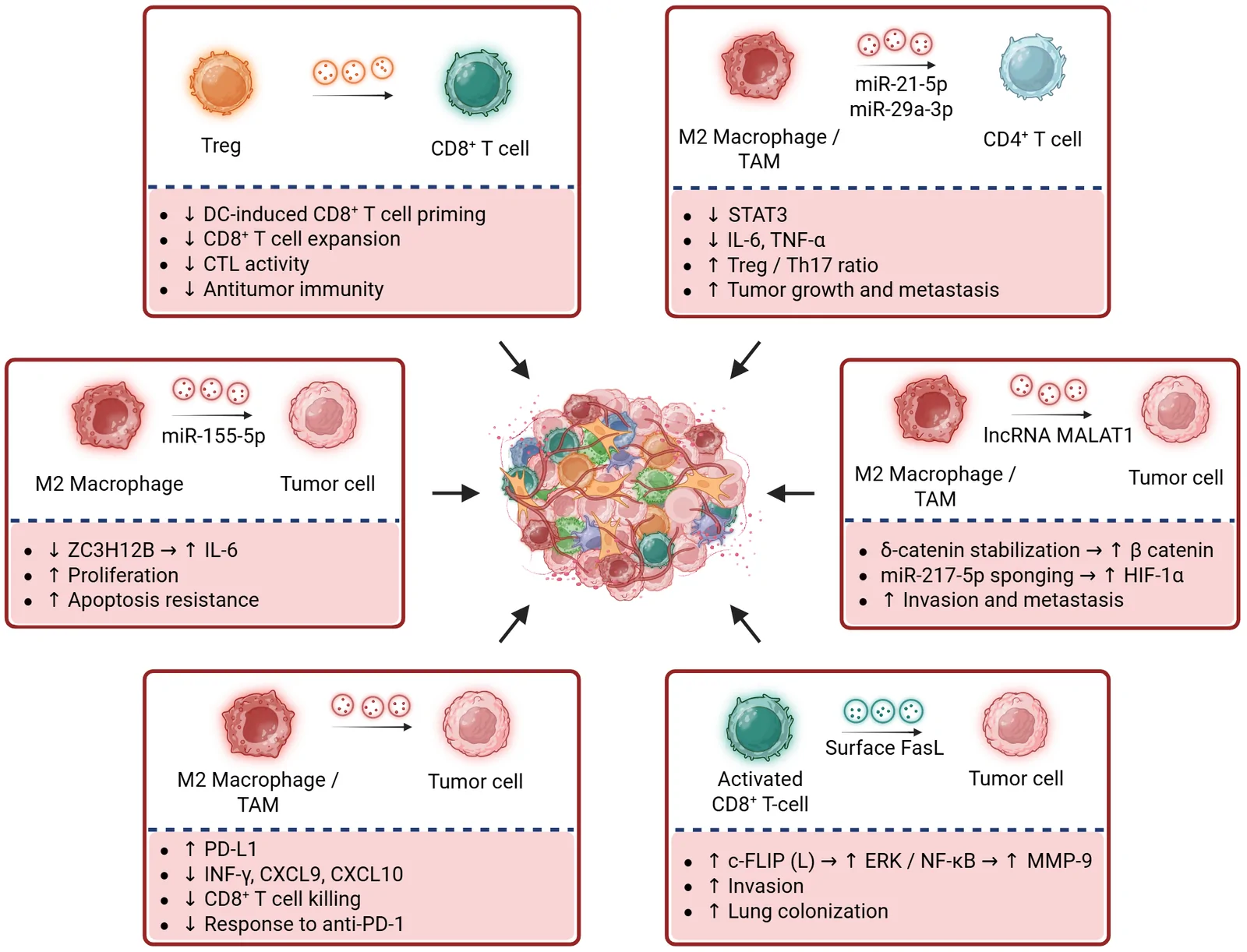
Representative protumor and immunosuppressive mechanisms of immune cell-derived exosomes. Immune cell-derived exosomes can promote immune suppression, tolerogenic reprogramming, cytokine-linked and checkpoint-mediated immune escape, tumor-promoting signaling, metabolic reprogramming, and metastatic progression. Collectively, these mechanisms weaken antitumor immunity and support tumor growth, invasion, metastasis, and treatment resistance.

Taken together, immune-cell-derived exosomes can promote tumor progression through cargo-dependent reprogramming of tumor and immune compartments, including effects on checkpoint signaling, cytokine regulation, metabolism, survival, and invasive behavior (**Figure 4**). However, the strength of mechanistic evidence varies across studies, and pathway changes should not be interpreted as proof of a causal cargo-recipient relationship without direct cargo perturbation and appropriate functional validation. Studies in section 4 are summarized in **Table 2**.

**Table 2:** Tumor-promoting and immunosuppressive mechanisms of immune cell-derived exosomes in cancer.

Protumor mechanism	Immune-cell source and vesicle type	Key cargo or feature	Primary recipient/target cell	Main target/pathway	Main protumor or immunosuppressive effect	Cancer type	Ref.
Immune suppression, tolerogenic reprogramming, and cytokine-linked immune escape	Activated CD4^+^CD25^+^FoxP3^+^ Treg-derived EVs	miR-150-5p and miR-142-3p	Dendritic cells	miRNA-mediated tolerogenic cytokine reprogramming	↑ IL-10 and ↓ IL-6 following; induction of a more tolerogenic DC phenotype	Not cancer-specific	(82)
	CD4^+^CD25^+^FoxP3^+^ Treg-derived exosomes	Surface CD73	CD4^+^ effector T cells	CD73-mediated conversion of extracellular AMP to adenosine; CTLA-4-independent suppression	↓ CD4^+^ T-cell proliferation; ↓ IL-2 and IFN-γ production	Not cancer-specific	(83)
	Natural CD8^+^CD25^+^ regulatory T-cell-derived exosomes	Treg associated markers	CD8^+^ T cells undergoing DC-mediated activation	↓ DC-cell-induced CD8^+^ T-cell priming	Suppression of DC-induced CD8^+^ T-cell priming and expansion; ↓ CTL activity; ↓ protective antitumor immunity	Melanoma	(84)
	M2/TAM-derived exosomes	miR-21-5p and miR-29a-3p	CD4^+^ T cells	miR-21-5p/miR-29a-3p-mediated targeting and ↓ STAT3	↑ Treg/Th17 ratio, IL-10, and TGF-β; ↓ IL-6 and TNF-α; ↑ tumor growth and metastasis	Epithelial ovarian cancer	(87)
	M2 macrophage-derived exosomes	miR-155-5p	Colon cancer cells; indirectly T cells	Direct targeting and ↓ ZC3H12B, reduced IL-6 mRNA degradation, ↑ IL-6	↑ Tumor-cell proliferation and apoptosis resistance; ↓ CD3^+^ T-cell proliferation and IFN-γ^+^ T-cell responses; ↑ immune escape	Colon cancer	(89)
Checkpoint-linked immune escape	Exosomes from IL-4/IL-13-polarized M2 macrophages	Specific cargo not identified	Gastric cancer cells	↑ p38 MAPK phosphorylation and ↑ PD-L1	↑ Tumor-cell proliferation and migration; ↓ apoptosis; promotion of PD-L1-associated immune escape	Gastric cancer	(91)
	M2-polarized TAM-derived exosomes	Specific cargo responsible for PD-L1 induction not identified	Cervical cancer cells	↑ Tumor-cell PD-L1 expression	↓ CD8^+^ T-cell killing and infiltration; ↓ IFN-γ, CXCL9, and CXCL10; ↑ tumor growth; ↓ response to anti-PD-1 therapy	Cervical cancer	(92)
Tumor-promoting signaling, metabolic reprogramming, and metastatic progression	M2 macrophage-derived exosomes used as a model of TAM-derived vesicles	ApoE protein	Gastric cancer cells	↑ PI3K-AKT-mTOR signaling, EMT-associated proteins, and actin cytoskeletal remodeling	↑ Migration, invasion, tumor-cell motility, and lung metastasis	Gastric cancer	(39)
	M2 macrophage-derived exosomes	miR-155-5p	NSCLC cells (H1299 and A549)	↑ VEGFR2 expression, activation of VEGFR2/PI3K-AKT-mTOR signaling	↑ Proliferation, migration, EMT, tumorigenicity, and xenograft tumor growth	Non-small-cell lung cancer	(95)
	HMC-1 mast-cell-derived exosomes	KIT protein; not c-KIT mRNA	Lung adenocarcinoma cells	KIT-SCF-linked signaling; ↑ phosphorylation of PI3K, AKT, and GSK3β; ↑ cyclin D1	↑ Tumor-cell proliferation and migration	Lung adenocarcinoma	(96)
	M2 TAM-derived exosomes	lncRNA MALAT1	Gastric cancer cells	Stabilization of δ-catenin, ↑ β-catenin signaling; miR-217-5p sponging, ↑ HIF-1α; ↑ glycolytic reprogramming	↑ proliferation, migration, invasion, tumor growth, and metastasis; ↑ oxaliplatin resistance	Gastric cancer	(97)
	Activated CD8^+^ T-cell-derived exosomes	Surface FasL	Fas-resistant melanoma and lung carcinoma cells	↑ c-FLIP(L), ↑ ERK and NF-κB, ↑ MMP9	↑ Tumor-cell invasion and lung colonization	Melanoma and lung carcinoma	(40)

↑ indicates increased/upregulated expression or activity.

↓ indicates decreased/downregulated expression or activity.

## 5 Integrated signaling networks regulated by immune cell-derived exosomes in the tumor microenvironment

The signaling pathways regulated by immune cell-derived exosomes do not operate as isolated molecular events. Their effects can extend across signaling pathways and cellular compartments, linking tumor-intrinsic responses with broader changes in immune activity within the tumor microenvironment.

PI3K-AKT signaling emerges as a shared node targeted by immune cell-derived exosomes from different sources. NK cell-derived exosomes suppress this pathway in tumor cells and link its inhibition to reduced PD-L1 expression and enhanced CD8^+^ T-cell activity, whereas M2 macrophage-derived exosomes activate PI3K-AKT signaling through distinct cargos, including ApoE and miR-155-5p, to promote tumor progression (39, 69, 95). These studies illustrate how a common signaling pathway can connect exosome-mediated regulation of tumor-cell behavior with broader immune effects in the tumor microenvironment.

A second pattern involves cytokine-linked communication between tumor and immune compartments. TAM-derived exosomal miR-21-5p and miR-29a-3p act directly on CD4^+^ T cells through STAT3, altering the Treg/Th17 balance and associated cytokine production. In contrast, M2 macrophage-derived exosomal miR-155-5p acts first on tumor cells through the ZC3H12B-IL-6 axis, with subsequent effects on T-cell responses (87, 89). Together, these studies illustrate two routes by which macrophage-derived exosomal cargo can reshape immune regulation: directly by altering immune-cell signaling and cytokine output, or indirectly by modifying cytokine signaling in tumor cells that then influences lymphocyte activity.

Another form of coordination occurs when a single exosomal cargo regulates parallel signaling routes within the same recipient cell. M2 TAM-derived exosomal MALAT1 simultaneously supports δ-catenin/β-catenin signaling and miR-217-5p/HIF-1α signaling, with both pathways contributing to glycolytic reprogramming and tumor progression (97). This provides an example of exosomal cargo coordinating parallel signaling pathways that converge on a common metabolic phenotype. Recipient-cell state can also redirect the biological outcome of exosomal signaling. In Fas-resistant tumor cells, FasL carried by activated CD8^+^ T-cell-derived exosomes did not trigger apoptosis but instead engaged c-FLIP(L), ERK, and NF-κB signaling, leading to MMP9-associated invasion (40). This illustrates how an exosomal signal can be routed toward a different biological outcome depending on the signaling competence of the recipient cell.

Together, these studies indicate that immune cell-derived exosomes can integrate signaling across shared pathway nodes, distinct cellular compartments, and parallel molecular routes. Importantly, the available evidence more commonly supports functional coordination than direct biochemical crosstalk among these pathways, with exosome-mediated signaling converging on immune regulation, metabolism, migration, and other tumor-associated phenotypes.

## 6 Challenges and future directions

Despite the growing promise of immune cell-derived exosomes in cancer immunotherapy and diagnostics, several translational challenges remain, including EV heterogeneity and characterization, scalable manufacturing, biodistribution, quality control, regulatory requirements, and clinical validation.

A primary challenge arises from the inherent heterogeneity of extracellular vesicle preparations. EVs can vary in size, surface composition, and nucleic-acid cargo, and bulk analyses may obscure functionally distinct subpopulations. Single-EV imaging combined with computational analysis can identify architectural heterogeneity, including distinct EV morphologies, that may be obscured in bulk measurements (100).

Isolation and characterization workflows further influence the interpretation and reproducibility of EV studies. Differential ultracentrifugation (UC), size exclusion chromatography (SEC), and immunoaffinity capture use distinct separation principles and can yield EV-enriched preparations with different recoveries, compositions, and levels of co-isolated material. Differential ultracentrifugation is widely used and can accommodate relatively large sample volumes, but it may co-isolate protein aggregates and other non-vesicular particles. SEC is comparatively gentle and can improve separation from soluble proteins, although it may dilute samples and does not necessarily resolve similarly sized contaminants. Immunoaffinity capture selectively isolates EV subtypes on the basis of surface markers and can provide high-purity preparations; however, it generally has low total-EV yield and high cost (5, 101).

These methodological differences can alter the apparent cargo profile, EV dose estimate, and measured biological activity. Therefore, direct comparison of functional outcomes across studies requires transparent reporting of donor-cell culture conditions, source material, isolation workflow, dose normalization, and complementary EV characterization (5). To overcome challenges related to sample complexity and reporting inconsistency, research in this field must rigorously align with updated methodological standardization and EV characterization guidelines, such as the Minimal Information for Studies of Extracellular Vesicles (MISEV) frameworks. Consistent with MISEV2023, EV preparations should be characterized using complementary approaches that assess particle properties, EV-associated components, and potential non-vesicular contaminants rather than relying on a single marker or analytical method (5).

Large-scale production remains another challenge for the clinical translation of exosome-based therapies. Conventional monolayer culture has limited capacity for high-density cell expansion, whereas 3D culture systems can support larger-scale cell growth and may increase EV production. Importantly, 3D culture conditions can also alter EV composition and biological activity compared with conventional monolayer culture; these changes may be therapeutically advantageous but must be carefully characterized and controlled to ensure reproducible EV products (102). Therefore, scalable manufacturing strategies must increase EV yield while preserving vesicle identity, composition, and function across batches. This will require standardized cell-culture conditions, collection and purification procedures, characterization methods, and mechanism-relevant potency testing (103, 104).

Maintaining EV stability after production is another important consideration for clinical translation. Repeated freeze-thaw cycles may alter vesicle structure and biological activity. Saccharide stabilizers, including sucrose, trehalose, and glucose, have been investigated as cryoprotectants that show promise in preventing membrane degradation and vesicle aggregation during storage. However, optimal storage conditions may differ depending on the cellular source and intended application of the vesicles (3, 105, 106).

Beyond production and storage, successful therapeutic use also depends on the *in vivo* fate of administered EVs. Following systemic administration, unmodified EVs can be cleared rapidly by the mononuclear phagocyte system and may accumulate in the liver and spleen; however, their biodistribution varies with vesicle source, administration route, dose, labeling strategy, and recipient physiology (107, 108). Real-time monitoring of EV biodistribution remains technically difficult because conventional lipophilic dyes may dissociate from vesicle membranes or remain in tissues after the vesicles have been cleared, potentially generating misleading signals. To overcome these limitations, more advanced approaches, including genetic reporter systems, metabolic radiolabeling, and PET/SPECT-compatible tracers, are being developed for more accurate, non-invasive tracking of EV pharmacokinetics (108). Improved imaging and tracking strategies are needed to determine where immune cell-derived exosomes accumulate, which cells take them up, and how their distribution affects therapeutic activity (104).

Collectively, these considerations highlight the need for well-defined quality-control criteria for therapeutic EV products, including identity, purity, particle quantity, composition, process-related contaminants, stability, and batch-to-batch consistency (109, 110).

Potency assessment remains challenging because EV activity can be evaluated using *in vitro* assays and *in vivo* disease models, which provide complementary but not directly interchangeable information. *In vitro* assays offer controlled, quantitative measurements of mechanism-relevant activity, whereas *in vivo* models provide disease-relevant assessment of therapeutic effects but introduce additional biological and procedural variability. Differences in EV source, isolation method, dose-normalization strategy, biodistribution, disease model, and experimental conditions can influence therapeutic outcomes. Consequently, a given EV dose does not necessarily predict a consistent therapeutic effect across EV preparations or experimental settings. Future development should prioritize validated, mechanism-relevant potency assays, well-defined dose metrics, dose-response testing, and batch-comparability assessment across production runs and laboratories (110).

Meeting these quality-control and potency requirements is central to the regulatory development of EV-based therapeutics. In the United States, exosome products intended for treatment are regulated as drugs and biological products and are subject to applicable premarket review; no exosome product is currently FDA-approved (111). Regulatory translation of EV-based therapies remains challenging because existing frameworks can be interpreted and applied differently across jurisdictions. EV products may also fall into regulatory gray areas depending on how they are classified, how extensively they are modified, and their intended use. Other uncertainties include defining EV-specific critical quality attributes, selecting suitable potency strategies when the mechanism of action is not fully understood, and determining comparability requirements after manufacturing changes. Early scientific advice and greater international harmonization may help support more consistent clinical translation of EV-based products (112).

Despite these translational challenges, immune cell-derived EVs are also being actively developed as therapeutic platforms. Engineering strategies may enhance their targeting, cargo delivery, and immunomodulatory functions. One emerging application is the use of engineered EV-based systems to deliver immune-checkpoint modulating agents. Immune-checkpoint inhibitors targeting pathways such as PD-1/PD-L1 and CTLA-4 have led to important clinical advances, but limitations such as immune-related adverse effects, off-target delivery, and limited response rates have remained (54, 57). Engineered EV-based systems may enable checkpoint-modulating agents to be combined with additional immunomodulatory cargo and delivered to tumors or immune cells involved in antitumor responses. For instance, macrophage-derived exosome-mimetic nanovesicles co-delivering the CD73 inhibitor AB680 and an anti-PD-L1 antibody promoted CD8^+^ T-cell responses and therapeutic activity in bladder cancer models. OVA-loaded dendritic cell-derived exosomes functionalized with anti-CTLA-4 antibodies also enhanced T-cell activation, increased the cytotoxic T-cell-to-regulatory T-cell ratio, and elevated antitumor cytokines such as IFN-γ and TNF-α (113–115).

Some immune cell-derived exosome approaches have also progressed to early clinical evaluation. Phase I studies in metastatic melanoma and advanced non-small-cell lung cancer demonstrated the feasibility of producing autologous dendritic-cell-derived exosome preparations and generally reported acceptable tolerability. A subsequent Phase II maintenance-immunotherapy study in advanced non-small-cell lung cancer showed enhancement of NK-cell activity but did not establish broad clinical efficacy. These studies support the feasibility of immune-cell-derived exosome administration while also highlighting the need for better-defined potency, dosing, manufacturing, and patient-selection strategies before broader clinical translation (116–118).

Overall, clinical development of therapeutic EVs is still in its infancy. Although initial studies have generally supported feasibility and acceptable tolerability, progression to later-phase efficacy trials have been limited. This underscores the need for more standardized dosing strategies, validated potency measures, and better-defined patient-selection criteria before immune cell-derived EV therapies can be evaluated more broadly (119).

Finally, computational and artificial-intelligence-based approaches may help address several of these translational challenges by improving the assessment of EV heterogeneity and interpretation of multidimensional EV datasets (100, 120, 121). Computational analysis of single-EV imaging can reveal heterogeneity that may be obscured by bulk measurements, while machine-learning approaches can support the analysis and classification of complex EV datasets (100, 120). For example, machine-learning classifiers have been used to distinguish Raman and FTIR spectral profiles of serum-derived EVs from patients with cancer and healthy controls (122). At the manufacturing and quality-control stages, machine-learning approaches may support assessment of EV size, concentration, surface-marker expression, and sample purity, therefore streamlining quality-control analysis (120). In addition, the Domain-Adaptive Diffusion Autoencoder for extracellular vesicles (DADA-EV) may estimate the relative tissue- and cell-type contributions to mixed EV transcriptomic profiles, supporting interpretation of complex circulating EV populations (121). Integrating computational tools with standardized production and characterization workflows may therefore improve interpretation of EV heterogeneity and support the development of more reproducible therapeutic preparations; however, computational predictions should be validated using independent datasets and EV preparations (120, 121).

## 7 Conclusion

Immune-cell-derived exosomes act as context-dependent regulators of tumor immunity and cancer progression. Across the studies reviewed, their biological effects depend on the interactions among donor-cell activation state, vesicular cargo, recipient-cell identity, and tumor-microenvironment conditions. Although many studies report changes in inflammatory, metabolic, checkpoint, or survival pathways after exosome treatment, the strength of mechanistic evidence is uneven because the specific cargo-target-pathway relationships are often not fully established.

Future research should prioritize three areas. First, studies should establish causal cargo-recipient cell-pathway relationships by combining donor-cell cargo perturbation, recipient-cell target validation, pathway-specific intervention, and functional rescue experiments. Second, clinical translation will require standardized EV production, isolation, characterization, dose normalization, storage, biodistribution assessment, and potency assays aligned with the intended therapeutic mechanism. Third, more controlled comparative studies are needed to determine how donor-cell activation state, cargo, recipient-cell type, and tumor context influence exosome composition and function and thereby shape antitumor or protumor outcomes.

Computational and AI-based approaches may help integrate donor-cell state, cargo composition, recipient-cell identity, and pathway-response data into predictive models, but their outputs require validation using independent EV preparations and functional experiments. Addressing these priorities will help determine when immune-cell-derived exosomes should be inhibited because they support tumor progression and immune escape and when they can be harnessed or engineered as cancer therapeutics.
